# Temporary Submucosal Inferior Maxillary Antrostomy: A Modification of the Inferior Antrostomy

**DOI:** 10.7759/cureus.34530

**Published:** 2023-02-02

**Authors:** Saud Alromaih, Nouf Aloraini, Abdulaziz Alrasheed, Ahmad Alroqi, Saad Alsaleh

**Affiliations:** 1 Otolaryngology - Head and Neck Surgery, King Saud University, Riyadh, SAU

**Keywords:** maxillary sinusitis, antrochoanal polyp, middle meatus antrostomy, inferior meatal antrostomy, maxillary sinus

## Abstract

Complete removal of maxillary sinus pathology can be challenging in specific locations. In the past, the Caldwell-Luc procedure was used for maxillary sinus disease. Currently, the endoscopic middle meatal antrostomy (EMMA) approach is used. However, it can often be difficult to reach certain locations of lesions by EMMA alone, requiring an endoscopic inferior meatal antrostomy (EIMA), which has been reported in the literature to have numerous complications. Furthermore, multiple techniques have been suggested for a combined bi-meatal approach to remove such lesions. We present a case of a 17-year-old with a challenging antrochoanal polyp (ACP) location requiring EIMA. The patient underwent our modified technique of submucosal inferior antrostomy with mucosal flap with no observed intra-operative and post-operative complications. Maxillary sinus pathology can be challenging due to limited access to specific regions. In this case report, we present a novel technique to achieve a temporary inferior antrostomy through a minimally invasive approach with a promising post-operative course.

## Introduction

The common approach to maxillary sinus pathology is the endoscopic middle meatal antrostomy (EMMA). This method gained acceptance in the 1980s when Messerklinger outlined the concept of the natural ostium [1]. This observation led to the widespread application of functional endoscopic sinus surgery (FESS), replacing the Caldwell-Luc procedure that used a transoral-transcanine approach to the maxillary sinus [2]. However, it is well known that maxillary sinus pathologies can be challenging to reach using the EMMA approach alone, mainly in lesions on the anteroinferior or anteromedial part of the maxillary sinus [3]. Endoscopic inferior meatal antrostomy (EIMA) is combined with EMMA to approach anatomically challenging sinus pathologies. However, this approach increases the risk of the recirculation phenomenon, in which mucus recirculates between adjacent openings into the maxillary sinus, leading to the recurrence of the disease [4]. In this case report, we present a modified technique of EIMA that should reduce the occurrence of the recirculation phenomenon.

## Case presentation

**Figure 1:**
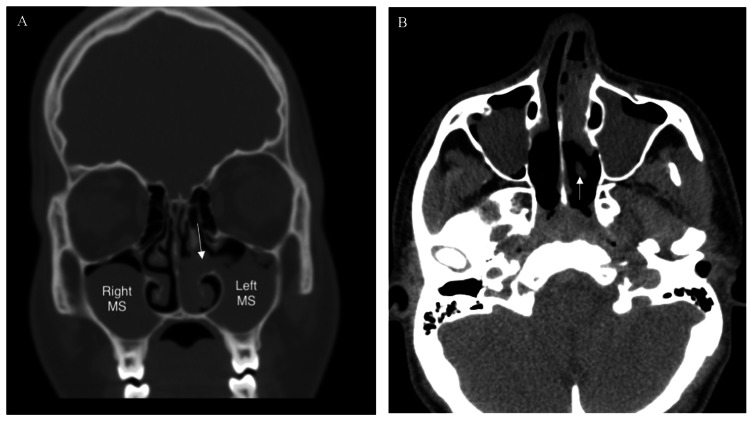
Pre-Operative Imaging. (A) Coronal CT of paranasal sinus showing multiple polypoidal densities occupying the maxillary sinuses bilaterally. (B) Axial CT of the same patient. Arrows: Left antro-choanal polyp S: maxillary sinus.

A healthy 17-year-old male presented to our clinic with a one-year history of nasal obstruction, which tended to occur on the left side. He had frequent thick yellow nasal secretions, left-sided facial pain, intermittent eyelid swelling, mouth breathing, and snoring. However, there were no signs of hyposmia, visual or allergic symptoms. The patient had previously used intranasal corticosteroids with no satisfactory improvement. A nasal endoscopic examination revealed a mild deviation of the nasal septum to the right side, bilateral inferior turbinate hypertrophy, and a nasal polyp occupying the left nasal cavity arising from the lateral nasal wall. The rest of the clinical exam was unremarkable, including his cranial nerves and orbital exam. The rest of the clinical exam, including his cranial nerves and orbital exam, were unremarkable. Computed tomography (CT) of the paranasal sinuses showed antrochoanal polyp (ACP) on the left nasal cavity and bilateral maxillary mucosal thickening with a large mucous retention cyst in the right maxillary sinus (Figures 1A, 1B).

**Figure 2:**
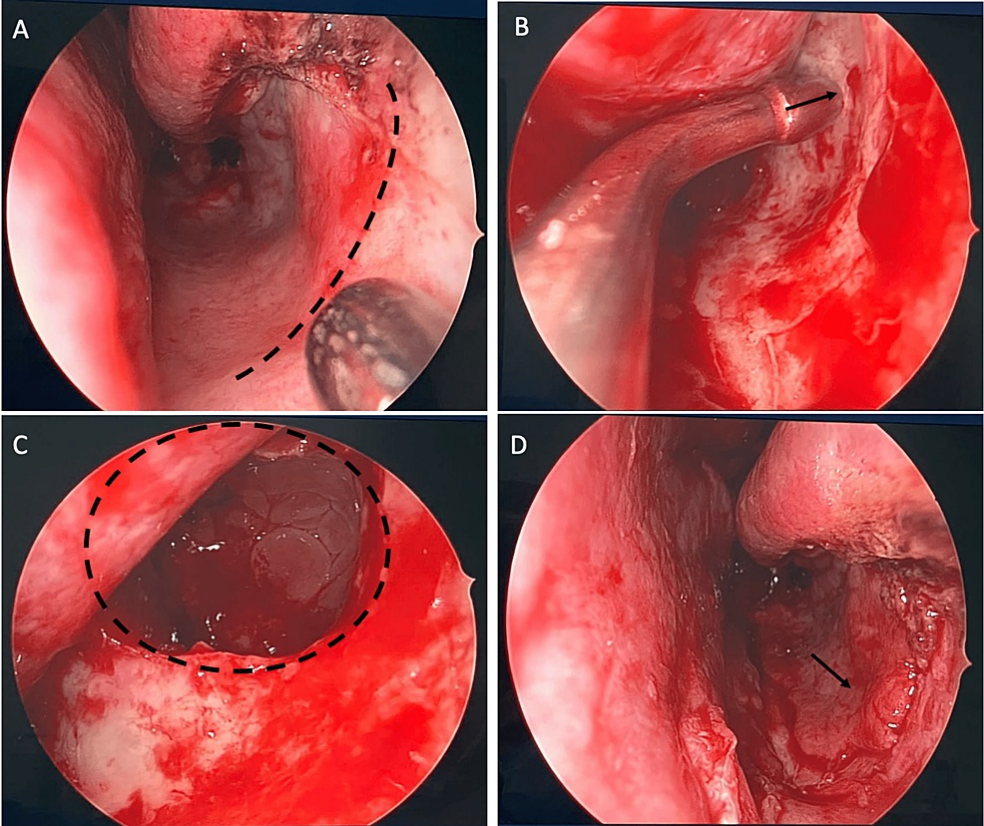
Intraoperative Photos. (A). Site of incision. (B) Submucosal inferior antrostomy site. (C) After widening the submucosal antrostomy. (D) After draping the mucosa.

The patient was counseled for septoplasty, turbinoplasty, and FESS. All benefits, risks, and alternatives were explained to the patient to which he consented. The surgical approach for the ACP on the left side was to endoscopically remove the nasal and middle meatal part of it. Then, uncinectomy was performed using the retrograde swinging door technique, and a large middle meatal antrostomy was created. The remaining part of the polyp was removed from the maxillary sinus. However, it could not be removed entirely from the maxillary floor. Thus, an inferior antrostomy was done to allow better intraoperative visualization and instrumentation. A sub-mucosal antrostomy was done to prevent future complications from the inferior antrostomy. Furthermore, a mucosal flap was raised from the nasal floor and the lateral wall of the inferior meatus. The incision was made at the level of the face of the inferior turbinate. Elevation of the mucosal flap was performed, followed by the inferior antrostomy. Angled scopes and instruments were used to ensure the complete removal of the polyp. Both zero and a 30-degree scopes were used to visualize maxillary sinus. The mucosal flap was then re-draped, and a small piece of the absorbable pack was applied around the mucosal incision (Figures 2A, 2D).

**Figure 3:**
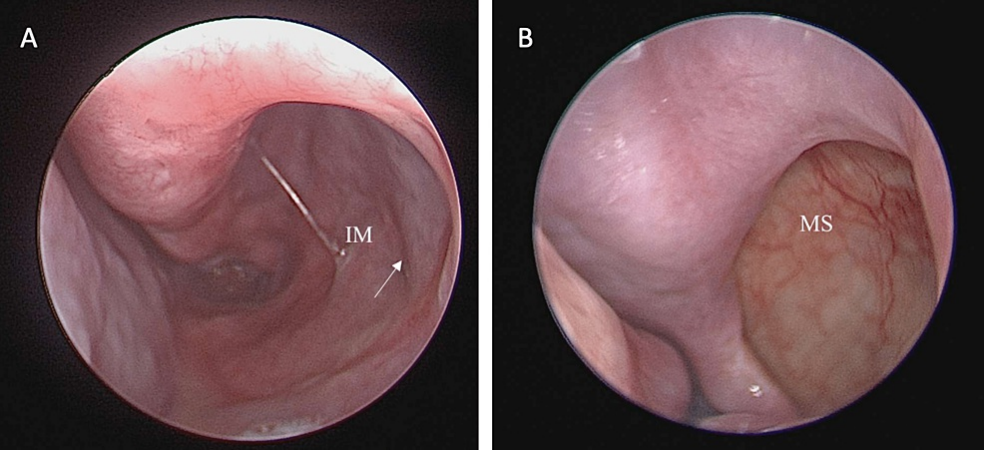
Post-operative result at one-year follow up. (A) Endoscopic image inferior meatus. (B) Endoscopic image middle meatus. Arrow: submucosal flap site. IM: inferior meatus, MS: maxillary sinus.

Post-operatively, the patient was prescribed intranasal corticosteroids and nasal irrigation then was discharged home. The patient was seen multiple times in the clinic during the first year, in which he remained symptom-free and had no postnasal drip/rhinorrhea, dryness, epiphora, dental complaint or neurological deficits. The endoscopic exam revealed a patent nasal cavity, and no synechia, crusting or recurrence of the disease, the mucosal flap of the inferior antrostomy healed well with complete closure and no sign of mucosal edema (Figures 3A, 3B).

## Discussion

The conventional inferior meatal antrostomy was abandoned in the modified Caldwell-Luc procedure due to increased morbidity without added benefits [5,6]. In some cases, the Cald-well-Luc procedure is not indicated, but a broad exposure is needed for full visualization and clearance of the sinus pathology such as maxillary fungal sinusitis to expose all sinus walls; EIMA would be the optimal option for such cases [7]. Furthermore, few studies discussed the outcomes of the advantage of a combined bi-meatal approach compared to EMMA alone, and all suggested that the combined approach had better outcomes regarding the complete removal of certain pathologies [7,8].

Moreover, the EIMA approach has its disadvantages, as it was reported to disrupt mucociliary clearance, increase the surgical time, the risk to injure the nasolacrimal duct and increase the risk of bleeding from the sphenopalatine artery [9]. Multiple surgical approaches were suggested to minimize these complications. For example, Zhao et al. described a modified endoscopic meatal fenestration with a small mucosal flap and a bone window of 10 mm that is closed after clearing the sinuses by repositioning the flap back and placing an absorbable suture. Post-operatively, they reported an intact lateral nasal wall in all 32 patients with no disease recurrence, but mucosal edema was observed [10].

Choi et al. described a minimal inferior meatal antrostomy using a similar approach to ours by generating a mucosal flap and a minimal bone window (1cm). On the contrary, our approach with a larger window provides wider exposure and a different mucosal incision site from the bone window to improve the chance of closure. They evaluated the closure of the window in their sample of 21 patients, with 76% of them having complete closure of the EIMA window [8].

## Conclusions

In this report, we followed the case of one patient who had undergone a submucosal inferior antrostomy with no observed intra-operative or post-operative complications. Upon follow-up, there was no reported recurrence or re-opening of the window, and the patient was symptom-free up to one year after the procedure. Possible complications of this specific approach, apart from all the complications of FESS, include nasolacrimal duct injury due to the drainage of tears through the valve of Hasner and persistent antrostomy. These complications were avoided by placing the incision anterior to the Hasner valve and achieving relaxed edges of the mucosal flap for the latter complication. Moreover, the report is limited in size as it is a single-patient case report. A follow-up case series in the near future is warranted to further assess our findings. Maxillary sinus pathology can be a challenging entity due to the limited access to specific regions. In this case report, we present a modified technique to achieve a temporary inferior antrostomy, through a minimally invasive approach with a promising post-operative course.
